# Milk Replacer Supplementation Ameliorates Growth Performance and Rumen Microbiota of Early-Weaning Yimeng Black Goats

**DOI:** 10.3389/fvets.2020.572064

**Published:** 2020-11-03

**Authors:** Zhaoqing Han, Aoyun Li, Lulu Pei, Kun Li, Taihua Jin, Fukuan Li, Zhennan Wang, Shenjin Lv, Yongzhu Li

**Affiliations:** ^1^College of Agriculture and Forestry Science, Linyi University, Linyi, China; ^2^College of Veterinary Medicine, Huazhong Agricultural University, Wuhan, China

**Keywords:** Yimeng black goats, milk replacer, growth performance, rumen microbiota, early weaning

## Abstract

Increasing evidence has indicated the ameliorative effect of milk replacer supplementation in ruminants for regulating their early growth and rumen development. However, it is still unclear whether milk replacer supplementation has a beneficial role in the growth performance and rumen microbiota of Yimeng black goats (YBGs). Therefore, this study was performed to investigate the effects of milk replacer on growth performance and rumen microbiota of YBGs. Our results revealed that milk replacer supplementation could significantly improve the growth performance of YBGs. Additionally, the results of alpha and beta diversities indicated that there was no significant difference in richness and diversity between the control and milk replacer-treated YBGs. At the phylum level, *Bacteroidetes, Firmicutes*, and *Proteobacteria* were the most dominant phyla in all the samples at different stages. Moreover, the YBGs treated with milk replacer possessed a higher abundance of *Verrucomicrobia* than that in the control YBGs, while the level of *Actinobacteria* was obviously decreased. It is noteworthy that the abundance of *Proteobacteria* in the control YBGs was higher than that in the YBGs supplemented with milk replacer throughout the experiment. At the level of genus, the differences in the richness between control and milk replacer supplement YBGs were gradually observed. Compared with the control YBGs, the proportion of *Akkermansia, Veillonella, Anaerovibrio, Ruminococcaceae_NK4A214_group, Ruminococcus_1*, and *Ruminococcus_2* was increased in the YBGs treated with milk replacer, whereas *Turicibacter* was decreased. In conclusion, milk replacer supplementation may serve as a good applicant for ameliorating early YBGs development and rumen microbiota.

## Introduction

Yimeng black goats (YBGs), mainly found in Shandong province of China, are characterized by strong resistance and excellent adaptability (1). This breed is an important source of meat, leather, and wool for local herdsmen. Previous studies have shown that the meat of YBGs have an extremely high nutritional value (2, 3). However, the population of YBGs is worryingly small due to delayed growth and low kidding rate. Therefore, it is crucial for the development of the goat industry to improve the growth performance of goats and shorten the pregnancy cycle of ewe. Currently, early-weaning and milk replacer supplementation are considered as the primary method to increase ewe breeding efficiency and goat's growth performance (4).

Early-weaning not only reduces the postpartum convalescence of the ewe but also increases the reproductive efficiency and promotes the development of digestive organs and survival of goats (5). Additionally, it may help in reducing the breeding and production costs of goats (6). However, goats may suffer from psychological, physical, and immune stress due to changes in the composition and physical form of their feed after weaning (7, 8). Importantly, the resistance of goats to various diseases will also decrease, due to the loss of maternal immunoglobulins (9, 10).

Increasing evidence has signified the beneficial effect of milk replacer supplementation in early-weaning goats for regulating their growth performance and rumen development (11). Milk replacer is artificial milk made by replacing milk protein with non-milk protein based on the nutritional standards of breast milk (12). Milk replacer supplementation not only reduces ewe consumption and the empty pregnancy rate but also improves reproductive efficiency and shortens the reproductive cycle (13). In addition, supplementing with milk replacer can also improve the growth performance of goats and decrease the morbidity and mortality caused by deficiency of early nutrient intake (14). Recent studies indicate that milk replacer supplementation is beneficial for the development and establishment of rumen microbiota (15). Rumen microbiota plays a key role in rumen function and nutrient digestion (16). However, it remains to be determined whether milk replacer supplementation has an ameliorative role in the growth performance and rumen microbiota of YBGs. Therefore, the objective of the present study was to investigate the effects of milk replacer on growth performance and rumen microbiota of YBGs.

## Materials and Methods

### Ethics Statement

The animal experiments were performed under the approval of the Ethics Committee of the Huazhong Agricultural University (Permit No. 4200695757).

### Animals and Sample Collection

A total of 30 1-day-old YBGs (initial weight 1.84 ± 0.86 kg) were purchased from a commercial feedlot (Shandong Province, China). All the selected YBGs had a similar genetic background and the same immune procedure. The YBGs were equally divided into two groups (*n* = 15), i.e., the control group (B group) and milk replacer (Table 1) supplementation group (R group). The YBGs in the B group were fed with goat's milk throughout the experimental period, i.e., from day 1 to 75, whereas the YBGs in the R group were fed with goat's milk from days 1 to 10. Afterwards, the YBGs in the R group were compulsively weaned on day 10 and were then provided with milk replacer (2% of the body weight from days 10 to 45 and 1.5% of the body weight from days 46 to 75) each day. Both groups received *ad libitum* regular starter feed (Table 2) and water from day 15. In addition, all animals were reared for 75 days, and body weight and average daily weight gain in each group was recorded on day 10, 15, 25, 45, and 75. During the experiment period, three YBGs from each group were randomly selected and euthanized by injecting pentobarbital (25 mg/kg) on day 10, 15, 25, 45, and 75. After euthanization, the carcasses were placed in a natural position to minimize the potential contamination among the four compartments. Afterwards, each stomach chamber was tied off using cotton rope and transferred to sterilized brown paper. The content of the rumen was homogenized and then the content which weighed approximately 200 g was stored in sterilized tubes. The collected samples were snap-frozen using liquid nitrogen and stored at −80°C for further analyses.

**Table 1:** Nutrient levels of milk replacer (DM basis).

**Ingredients**	**Content (%)**	**Nutritional level**	**Content (%)**
Corn	43	DM (%)	97.58
Soybean meal	2.2	DE/(MJ /kg)	15.90
Wheat bran	6	CP (%)	24.80
Alfalfa	10	CP/DE (g/MJ)	15.60
Skim milk	10	EE (%)	15.43
Whey	10	Lactose (%)	14.52
Fish meal	2.5	Ash (%)	7.70
Sucrose	10	Calcium (%)	1.02
Nacl	0.3	Phosphorus (%)	0.66
Premix	4		
Total	100		

*DM, DE, CP, EE, and Ash indicate dry matter, digestible energy, crude protein, ether extract, and crude ash, respectively. Nutrient levels were measured values except DE which were calculated values. The premix provided the following per kg of diets: VA 13,000 IU, VD3 000 IU, VE 70 IU, Cu (as copper sulfate) 5–100 mg, Fe (as ferrous sulfate) 100 mg, Mn (as manganese sulfate) 80 mg, Zn (as zinc sulfate) 40 mg, I (as potassium iodide) 3.2 mg, and Se (as sodium selenite) 0.6 mg. For the ingredients of milk replacer refer to the invention patent ZL201210365927.6*.

**Table 2:** Composition and nutritional level of starters (DM basis).

**Ingredients**	**Content (%)**	**Nutritional level**	**Content (%)**
Corn	53	DM (%)	86.59
Soybean meal	27	DE/(MJ /kg)	14.07
Wheat bran	6	CP (%)	20.80
Premix	4	CP/DE (g/MJ)	13.90
Alfalfa meal	10	EE (%)	3.77
Total	100	Lactose (%)	14.52
		Ash (%)	8.52
		Calcium (%)	0.95
		Phosphorus (%)	0.70

*DM, DE, CP, EE, and Ash indicate dry matter, digestible energy, crude protein, ether extract, and crude ash, respectively. The premix provided the following per kg of diets: VA 12,000 IU, VD 2,000 IU, VE 30 IU, Cu (as copper sulfate) 12 mg, Fe (as ferrous sulfate) 64 mg, Mn (as manganese sulfate) 56 mg, Zn (as zinc sulfate) 60 mg, I (as potassium iodide) 1.2 mg, and Se (as sodium selenite) 0.4 mg. The composition and nutrient level of starters refer to previous research with some modifications (17)*.

### gDNA Extraction

Bacterial genomic DNA was extracted using a QIAamp DNA Mini Kit (QIAGEN, Hilden, Germany) following the producer's instructions. DNA quality was determined by electrophoresis on agarose gel 0.8% (w/v) and the quantity was determined using a Nanodrop™ spectrophotometer (NanoDrop Technologies, Thermo Scientific, USA).

### 16S rRNA Amplification

Specific PCR primers (338F: ACT CCT ACG GGA GGC AGC A and 806R: GGA CTA CHV GGG TWT CTA AT) based on conserved regions were synthesized to amplify the V3/V4 regions. An AxyPrep DNA Gel Extraction Kit (Axygen, CA, USA) and 2% agarose gel electrophoresis were used for target fragment recovery and PCR amplification product evaluation, respectively. The PCR amplification recovery products were quantified on a fluorescent Microplate reader (BioTek, FLx800) via a Quant-iT PicoGreen dsDNA Assay Kit based on the preliminary quantitative results of electrophoresis. For the sequence library construction, a TruSeq Nano DNA Low Throughput Library Prep Kit (Illumina, CA, USA) was used following manufacturer's specifications. Amplified products' sequence ends were repaired by End Repair Mix2. To enrich the sequencing library template, PCR amplification was carried out and the library enrichment product was purified again using BECKMAN AMPure XP Beads. The final fragment-selection and purification of the library was performed using 2% agarose gel electrophoresis.

The quality of the libraries was examined on an Agilent Bioanalyzer using the Agilent High Sensitivity DNA Kit prior to the sequencing procedure. The libraries with only one peak signal and no linker signal were considered for the procedure. Moreover, the libraries were quantified using a Quant-iT PicoGreen dsDNA Assay Kit on the Promega QuantiFluor fluorescence quantification system. The qualified library concentration must be above 2 nM. The qualified libraries were gradient diluted and mixed in proportion according to the amount of sequencing required. The MiSeq Reagent Kit V3 (600 cycles) was used to perform 2 × 300 bp paired-end sequencing on the MiSeq sequencing machine after the mixed libraries were denatured into single strands by sodium hydroxide. The original sequence data were submitted to the Sequence Read Archive (SRA) (NCBI, USA) with the accession no. PRJNA637829.

### Bioinformatics and Statistical Analysis

The QIIME software (Qiime1.9.1) was used for 16S rRNA original data quality preliminary screening and analysis. Interrogative and short sequences (<200 bp) were discarded using the QIIME software. The obtained sequences were clustered and OTU partitioned at ≥97% sequence similarity by using the clustering program VSEARCH (1.9.6.). A representative sequence of each OTU was classified at a confidence threshold of 0.8 according to the Ribosomal Database Program (RDP) classifier. Additionally, the obtained sequences with 97% similarity were merged to the same operational taxonomic units (OTUs). Before calculating alpha and beta diversity statistics, the sequencing depth of each sample was evaluated using sparse curves. Continuous analysis of alpha diversity and beta diversity were performed based on the output normalized date. Four metrics including Chao1, ACE, Simpson, and Shannon were used to analyze alpha diversity (18, 19). GraphPad Prism (version 6.0c) and R (v3.0.3) software were used for statistical analysis (20). The criterion of significance was performed at *p* < 0.05 and the values were presented as means ± SD.

## Results

### Effect of Milk Replacer Supplementation on Growth Performance of YBGs

**Figure 1:**
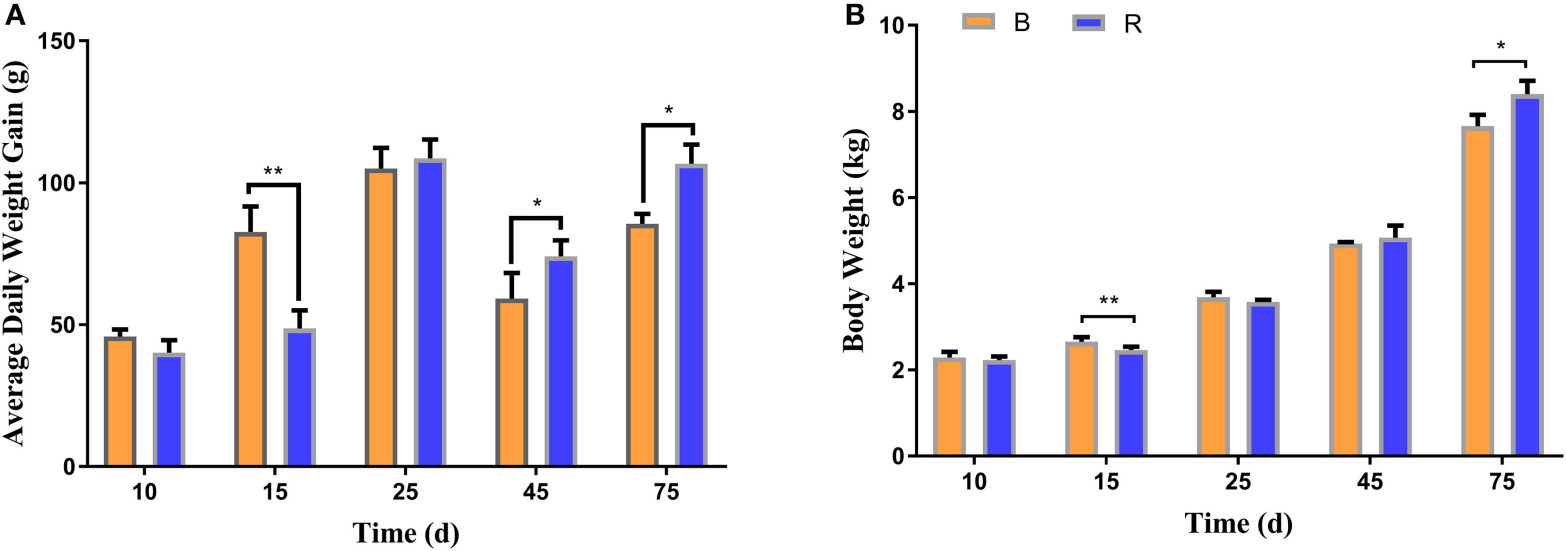
Milk replacer supplementation improved the growth performance of YBGs. **(A,B)** indicate average daily weight gain and body weight of goats on days 10, 15, 25, 45, and 75, respectively. B represents the YBGs in the control group, while R indicates the YBGs in the milk replacer supplementation group. The data are expressed as the mean ± SD. ^*^P < 0.05, ^**^P < 0.01.

To investigate the effect of milk replacer supplementation on the growth performance of YBGs, the body weight and average daily weight gain of both groups were recorded. The average initial body weight of YBGs in the B and R groups were 1.84 ± 0.073 kg and 1.85 ± 0.09 kg, respectively, with similar values. The body weight gain of the B group was higher than that of the R group from days 1 to 15, whereas the body weight of the B group was significantly higher than that of the R group on day 15 (*P* < 0.01) (Figure 1A). However, statistical analysis indicated that the average daily weight gain of the R group surpassed that of the B group after day 25, and a significant difference was observed on day 45 and day 75 of the experiment (*P* < 0.05) (Figure 1A). Additionally, the body weight differed significantly between the groups and reached 7.66 ± 0.26 kg and 8.40 ± 0.31 kg, respectively, on day 75 (*P* < 0.05) (Figure 1B).

### Sequences Analyses

**Figure 2:**
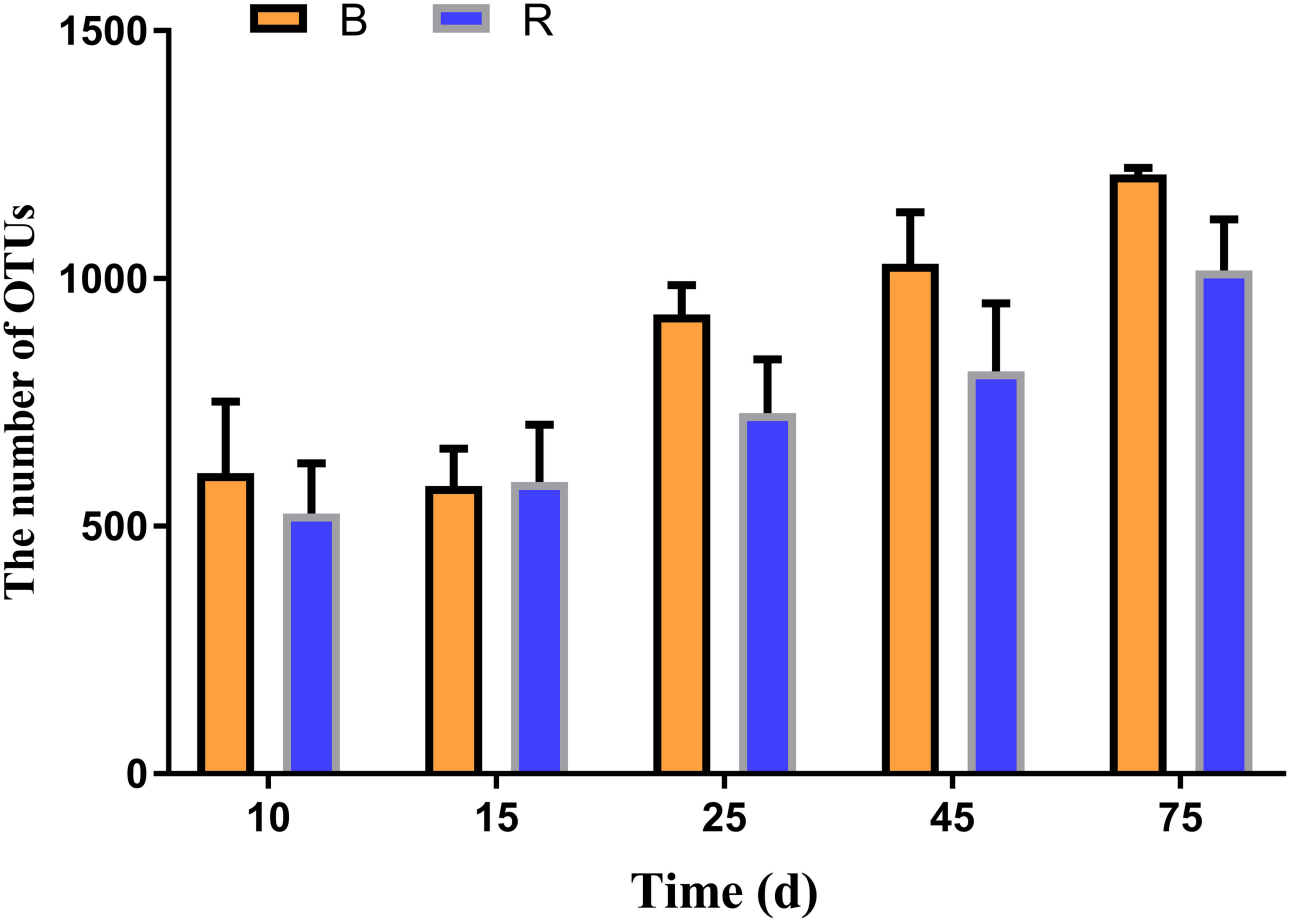
The number of OTUs in the B and R groups on day 10, 15, 25, 45, and 75. B represents the YBGs in the control group, while R indicates the YBGs in the milk replacer supplementation group.

In the current study, 30 rumen samples collected from goats were subjected to high-throughput sequencing analysis. After optimizing the preliminary data, a total of 877,614 and 883,962 high-quality valid sequences were obtained from the B and R groups, respectively. The rarefaction (Chao1 and Shannon) and rank abundance curves showed a relatively flat curve and a tendency to saturate characteristic (Supplementary Figures 1A–C). The high-quality sequences were merged and OTU partitioned based on 97% nucleotide sequence similarity. The average number of OTUs in the R and B groups gradually increased from days 10 to 15, respectively, and all the samples showed the highest number of OTUs on day 75 (Figure 2). Throughout the experiment, the number of OTUs in the B group were higher than that in the R group except on day 15, other than that no significant difference was observed between the two groups (Figure 2).

### Effects on Microbial Community Diversity

**Figure 3:**
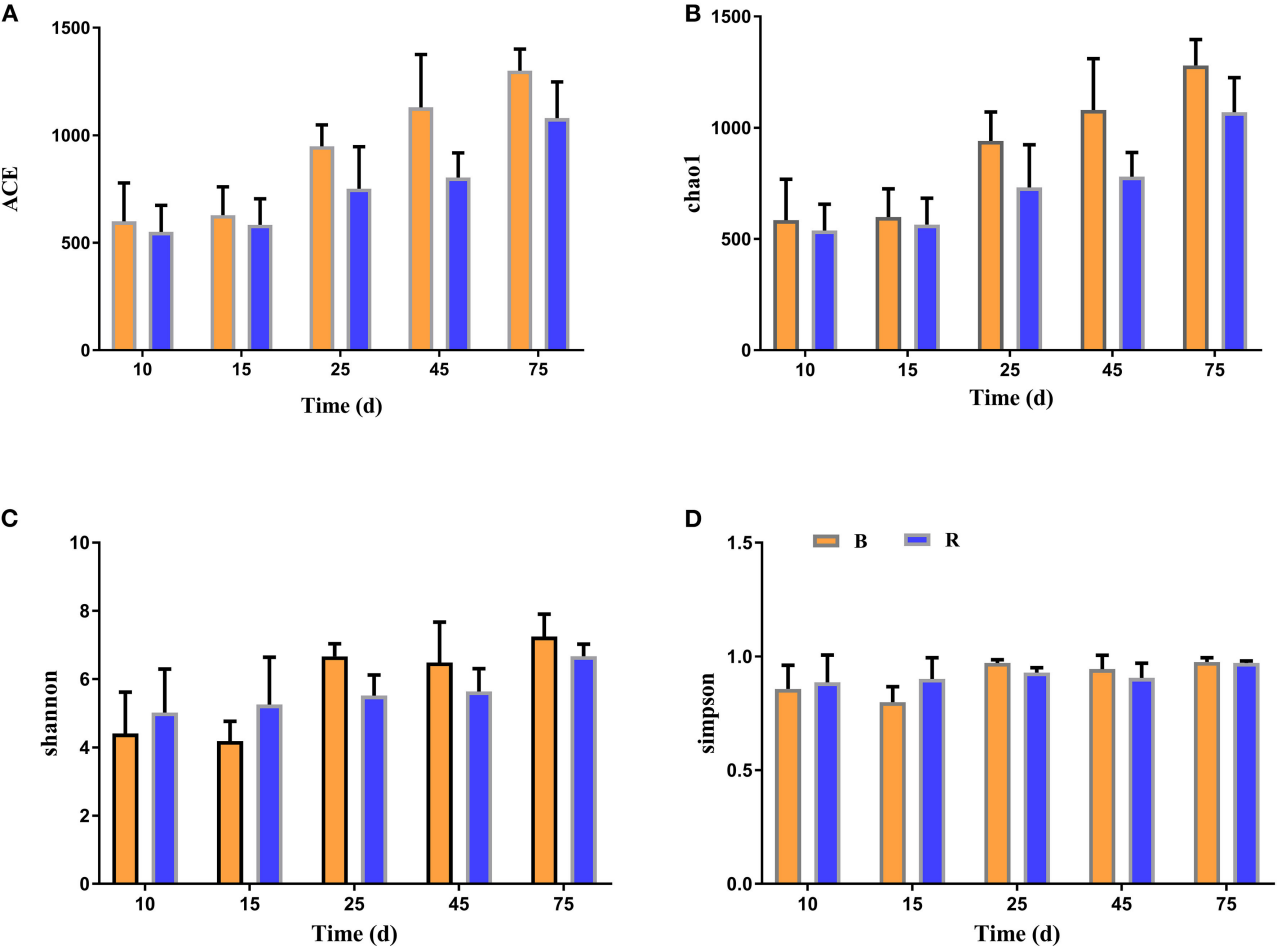
The diversity indices of rumen microbiota in different groups. Chao1 **(A)**, ACE **(B)**, Shannon **(C)**, and Simpson **(D)** were used to evaluate the alpha diversity of rumen microbiota. B represents the YBGs in the control group, while R indicates the YBGs in the milk replacer supplementation group.

**Figure 4:**
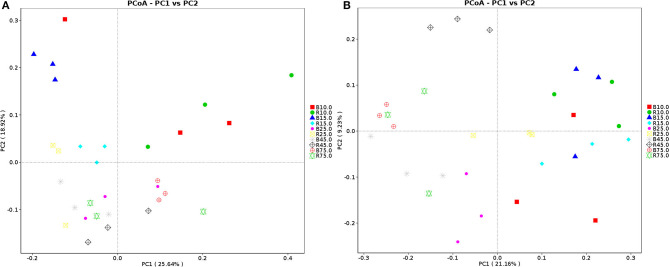
Principal coordinate (PCoA) analysis of rumen microbiota. **(A)** PCoA map based on weighted uniFrac distance; **(B)** PCoA map based on unweighted uniFrac distance. Each point indicates one sample. The distance of the two points indicate the difference of rumen microbiota. B represents the YBGs in the control group, while R indicates the YBGs in the milk replacer supplementation group.

The alpha diversity of rumen microbiota was evaluated using Chao1, ACE, Shannon, and Simpson. Good's coverage estimates were ~100% for all samples, showing excellent coverage. The ACE and Chao1 indices in all samples showed a gradually upward trend with the increase of experiment time. Specifically, the average of the ACE index in the B group varied from 636 to 1,437, while the Chao1 index varied from 608 to 1,386. Moreover, the average value of the ACE index in the R group ranged from 627 to 1335, while the average values of the Chao1 index ranged from 601 to 1,301. The ACE and Chao1 indices of the B group were higher than that of the R group throughout the experiment period, other than that no obvious difference was observed between the two groups (*P* > 0.05) (Figures 3A,B). The results of the Chao1 and ACE indices suggested that there was no significant difference in the rumen microbiota abundance between the two groups. Compared with the B group, the Shannon and Simpson indexes in the R group were relatively higher on days 10 and 15, but lower on days 25, 45, and 75. Statistical analysis showed that both B and R groups exhibited the highest Shannon (7.42 and 6.83) and Simpson (0.977 and 0.973) indices on day 75. However, there was no significant difference in the average of the Shannon and Simpson indexes between the two groups during the whole experimental period (*P* > 0.05) (Figures 3C,D). The Shannon and Simpson indices revealed a non-significant difference in the rumen microbiota diversity among all the samples. The results of beta diversity demonstrated that the samples in the B and R groups gradually clustered with time, suggesting that rumen microbiota was not different between experimental groups (Figures 4A,B).

### Composition Analysis of the Microbial Community Structure in Different Groups

**Figure 5:**
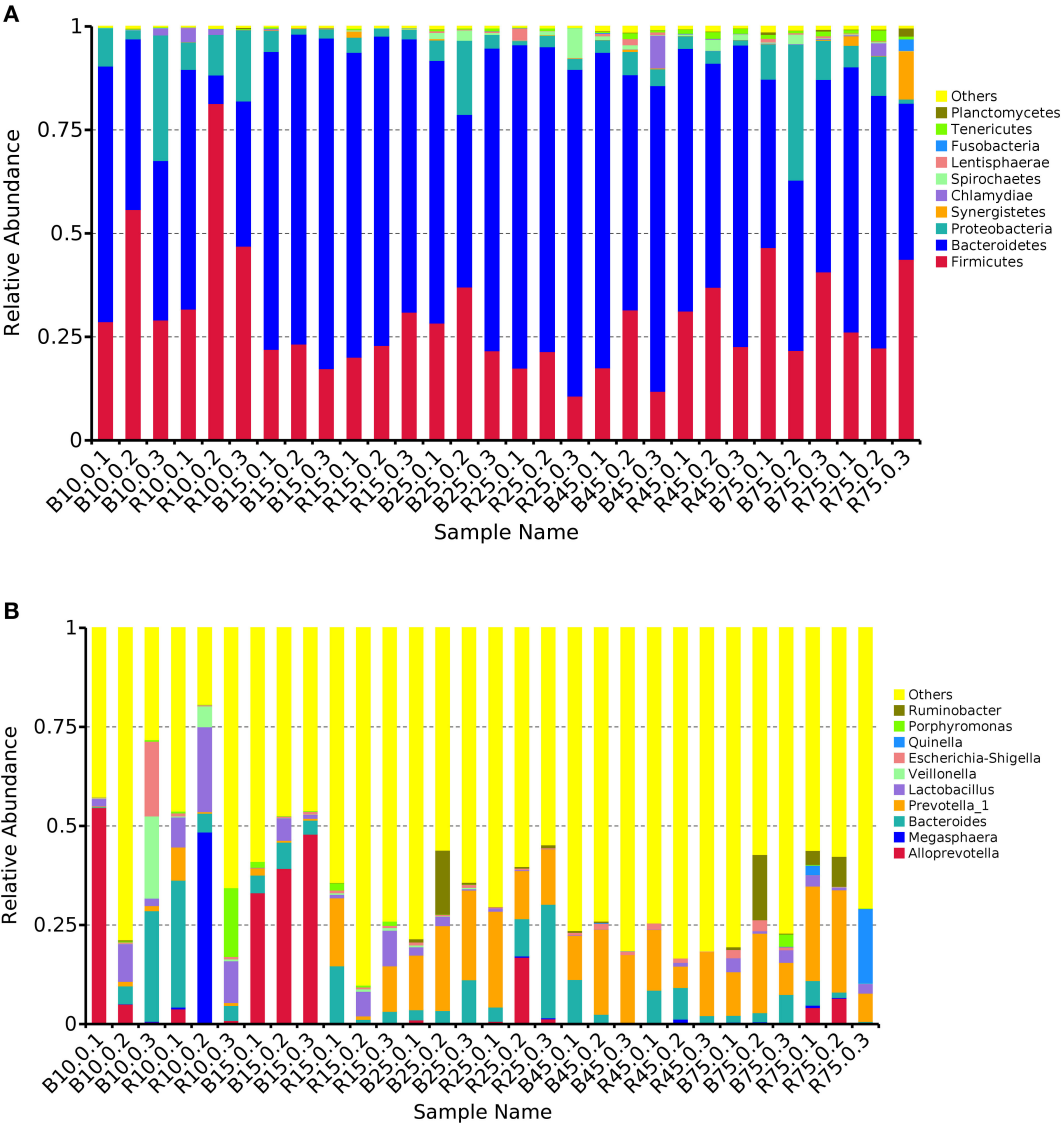
The relative abundance of microbial composition of different samples. **(A)** The top 10 dominant phylum of the rumen microbiota in the B and R groups. **(B)** The top 10 primary genera of the rumen microbiota in the B and R groups. B represents the YBGs in the control group, while R indicates the YBGs in the milk replacer supplementation group.

The rumen microbiota in the B and R groups were assessed at different taxonomical levels. The three most abundant (>90%) phyla in all samples were *Firmicutes, Bacteroidetes*, and *Proteobacteria* (Figure 5A). Other phyla including *Synergistetes, Chlamydiae, Spirochaetes, Lentisphaerae*, and *Fusobacteria* were present with a lower abundance accounting for <8% of the total 16S rRNA gene sequences in all samples. On day 10, *Bacteroides* (10.94%), *Veillonella* (7.00%), and *Escherichia-Shigella* (6.55%) were predominant in the B group, whereas *Megasphaera* (16.35%), *Bacteroides* (13.50%), and *Lactobacillus* (13.21%) were prominent in the R group at the genus level (Figure 5B). Additionally, the most abundant genera were *Alloprevotella* (40.84%) and *Bacteroides* (4.83%) in the B group, whereas *Bacteroides* (6.19%) and *Prevotella_1* (8.85) were predominant in the R group on day 15. Interestingly, *Bacteroides* and *Prevotella_1* were the dominant bacteria in the B and R groups on days 25, 45, and 75.

**Figure 6:**
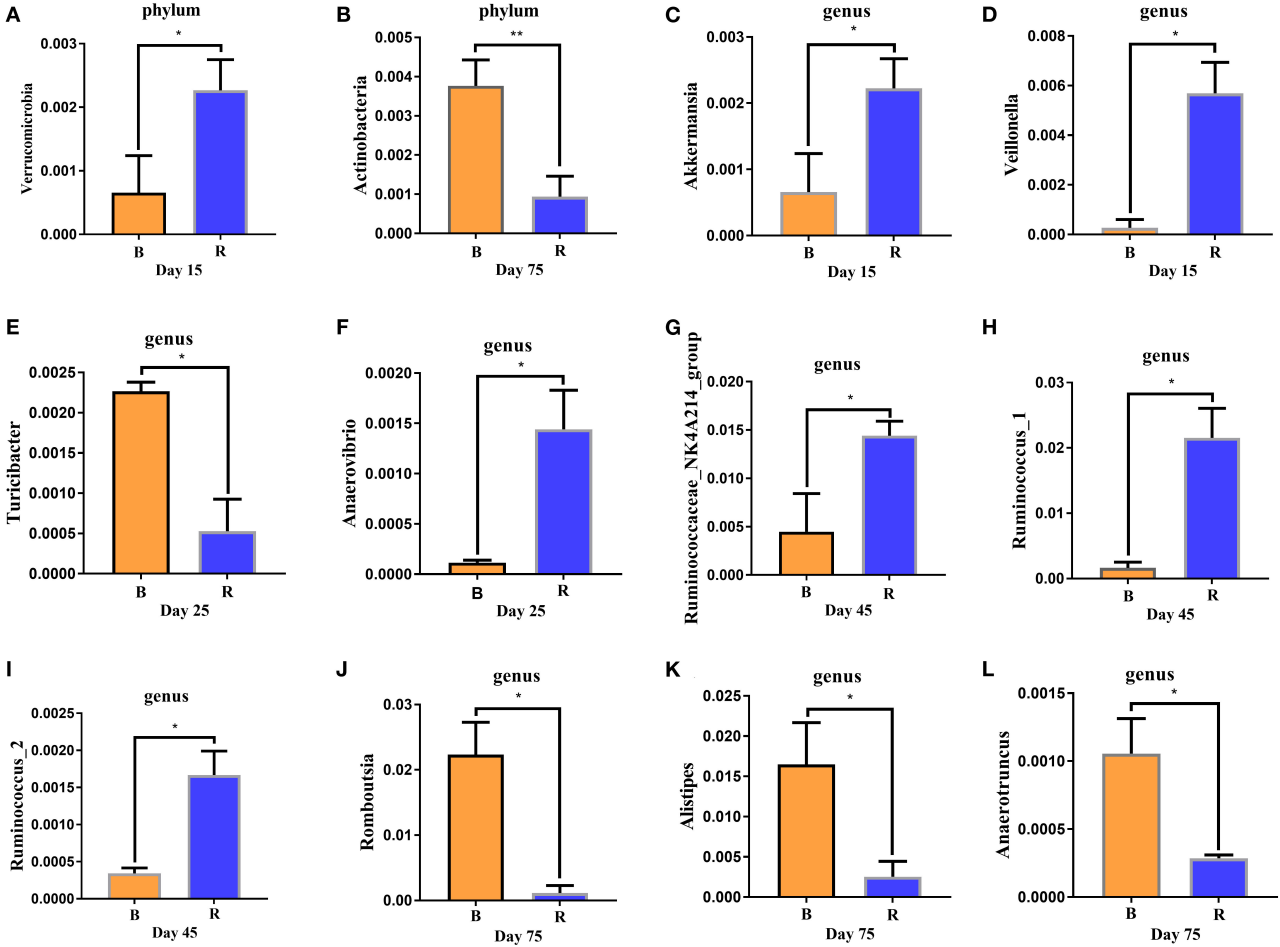
**(A–L)** Differences in rumen bacteria abundance between the B and R groups. B represents the YBGs in the control group, while R indicates the YBGs in the milk replacer supplementation group. The results were evaluated through one-way ANOVA. All of the data represent means ± SD. ^*^p < 0.05, ^**^p < 0.01.

The comparison results of rumen microbiota between the B and R groups using Metastats showed significant abundance of *Verrucomicrobia* (*P* < 0.05) in the R group when compared to the B group on day 15, while *Actinobacteria* (*P* < 0.01) was significantly lower on day 75 (Figures 6A,B). The abundance of *Proteobacteria* in the B group was found relatively higher, but it was not statistically significant (*P* > 0.05) when compared to the R group (data not shown). At the genus level, *Akkermansia* and *Veillonella* in the R group was significantly higher (*P* < 0.05) than the B group on day 15 (Figures 6C,D). Moreover, a comparison of the B and R groups showed an obvious decrease (*P* < 0.05) in the abundance of *Anaerovibrio* as well as a significant increase (*P* < 0.05) in the abundance of *Turicibacter* on day 25 (Figures 6E,F). The relative abundance of *Ruminococcaceae_NK4A214_group, Ruminococcus_1*, and *Ruminococcus_2* were higher (*P* < 0.05) in the R group than in the B group on day 45, whereas the abundance of *Romboutsia, Alistipes*, and *Anaerotruncus* in the R group were lower (*P* < 0.05) when compared to the B group on day 75 (Figures 6G–L).

## Discussion

Currently, milk replacer has been widely used in the livestock industry due to its stable quality and high nutritional value (21, 22). However, it remains to be determined whether milk replacer supplementation has protective and ameliorative roles in the growth performance and rumen microbiota of YBGs. In this study, we investigated the effect of milk replacer supplementation on the growth performance and rumen microbiota of YBGs on different growth days. The results indicated that milk replacer supplementation improved the growth performance of YBGs, whereas the rumen microbiota abundance and diversity between experimental groups were not different.

The adaptation of animals to feed replacement is a gradual process, especially in juvenile ruminants with an immature gastrointestinal tract (23). Therefore, the abrupt change of feed from udder milk to milk replacer can result in a severe stress response, which is also considered as an important factor restricting the growth of young animals (24). In this study, the stress response may be one of the reasons for the slow growth of YBGs in the R group from day 1 to 15. The active substances and nutrients in udder milk were easily affected by multiple external factors, which may hinder the growth and development of goats (25). Conversely, high-quality milk replacer provides comprehensive nutrients to goat growth to compensate for the deficiency of udder milk (26, 27). We observed that milk replacer supplementation gradually improved the growth performance of YBGs, which was consistent with the results of numerous previous studies (23, 28). Moreover, milk replacer can reduce the stress response caused by feed alterations, which contribute to the overall health of the body and rumen development.

Generally, *Proteobacteria, Bacteroidetes*, and *Firmicutes* were the most predominant phyla in the ruminants and the content of each phylum was dynamically diverse and influenced by many factors including feeding mode, animals' species, and feed (29, 30). Our results indicated that *Proteobacteria, Bacteroidetes*, and *Firmicutes* were the three most dominant phyla in the rumen microbiota of YBGs, which were consistent with previous observations in bovine, sheep, and yak (31, 32). Remarkably, the *Proteobacteria* level of the R group displayed a downward trend as compared to the B group. It is well-known that *Proteobacteria* is one of the largest phyla, that comprises gram-negative pathogenic bacteria including *Helicobacter pylori, Escherichia coli, Salmonella*, and *Vibrio cholerae* (33, 34). The higher abundance of *Proteobacteria* in the rumen microbiota may induce an immune response and increase the risk of pathogen infection in the host. Previous studies have suggested that the synergy between *Actinobacteria* and one partner or host can easily translate into pathogenic interactions with another (35). Furthermore, (36) observed that the abundance of *Actinobacteria* in the rumen of dairy cattle with subacute ruminal acidosis was significantly increased. Compared with nutritionally stable milk replacer, milk is easily influenced by the feeding environment and nutritional condition, which causes the decline of the quality and safety of the milk (26). Importantly, poor quality milk may alter the rumen microbiota in a negative way (26). In the current study, the R group had lower *Proteobacteria* and *Actinobacteria* content as compared to the B group, which signified that milk replacer supplementation may decrease the risk of disease and contribute to growth and rumen microbiota balance of early-weaned goats.

At the genus level, *Lactobacillus* was the predominant genera in the rumen of the R group on day 10, which differs from the B group. Previous research indicated that *Lactobacillus* plays an important role in maintaining rumen microbiota and improving the digestive power of rumen (37–40). Additionally, *Lactobacillus* can also inhibit the proliferation of pathogenic bacteria in the rumen by producing organic acid and antimicrobial peptides (39, 40). All the goats remained healthy, with no sign of acidosis, in the context of *Lactobacillus* increase. Compared with the B group, the R group had a higher *Lactobacillus* level, which may be one of the reasons for the rapid growth of YBGs in the R group. Remarkably, the percentage of *Akkermansia, Anaerovibrio, Veillonella, Ruminococcus*, and *Ruminococcaceae* in the R group was significantly increased, while the ratio of *Turicibacter* was obviously decreased compared with the B group. *Ruminococcus* mostly inhabits the rumen and hindgut of cud-chewing animals and contributes to the degradation of cellulose and starch (41). *Ruminococcus* can produce acetic acid, formic acid, and a small amount of lactic acid (42). Previous research has indicated that short-chain fatty acid including formic acid, acetic acid play a critical role in regulating the balance of gut microbiota and maintaining morphology and function of intestinal epithelial cells (43, 44). Moreover, the short-chain fatty acid regulates energy intake by the brain-gut axis (45). Lactic acid can improve digestive enzyme activity and exert bacteriostatic effects by regulating rumen pH (39). Furthermore, lactic acid supplementation in feed not only improves the growth performance of juvenile animals but also decreases the occurrence rate of gastrointestinal bacterial diseases (46–48). *Akkermansia* helps to maintain digestive tract health and reduce the risk of obesity, diabetes, and inflammation (49, 50). *Anaerovibrio* participates in the breakdown of fats and sugars and produces propionic acid, acetic acid, and succinic acid (51). *Veillonella* enhances the respiratory and digestive system immunity of the host and decreases the incidence of tooth decay (52, 53). Our results conveyed important information that milk replacer supplementation improved the rumen microbiota by increasing the ratio of beneficial and pathogenic bacteria.

In conclusion, the current study investigated the effect of milk replacer on the growth performance and rumen microbiota of early-weaning YBGs. The results indicated that milk replacer supplementation can improve the growth performance and rumen microbiota of YBGs. However, this study has some limitations such as incontrollable variables including individual variability caused by eating behavior and sample collection. However, our study revealed that milk replacer supplementation may have a beneficial role in improving growth performance and rumen microbiota during dysbacteriosis and weaning stress.

## Data Availability Statement

The datasets presented in this study can be found in online repositories. The names of the repository/repositories and accession number(s) can be found at: https://www.ncbi.nlm.nih.gov/, PRJNA637829.

## Ethics Statement

The animal study was reviewed and approved by the Ethics Committee of the Huazhong Agricultural University.

## Author Contributions

ZH, YL, and SL conceived and designed the experiments. AL, LP, KL, TJ, FL, and ZW contributed sample collection and reagents preparation. ZH, AL, and LP analyzed the data. ZH wrote the manuscript. ZH, AL, KL, and LP revised the manuscript. YL and SL provided resources. All authors reviewed the manuscript.

## Conflict of Interest

The authors declare that the research was conducted in the absence of any commercial or financial relationships that could be construed as a potential conflict of interest.

## Supplementary Material

Supplementary Figure 1 Feasibility analysis of different samples. Each curve represents a sample. The rarefaction curves **(A,B)** were used to evaluate the adequacy of sequencing for each sample and the Rank abundance curve **(C)** was used to assess the abundance and evenness of samples. B represents the YBGs in the control group, while R indicates the YBGs in the milk replacer supplementation group.
