# Supplementary figures and images for: Milk Replacer Supplementation Ameliorates Growth Performance and Rumen Microbiota of Early-Weaning Yimeng Black Goats
Source: Front Vet Sci. 2020 Nov 3;7:572064. doi: 10.3389/fvets.2020.572064 (PMC7669828; doi:10.3389/fvets.2020.572064)

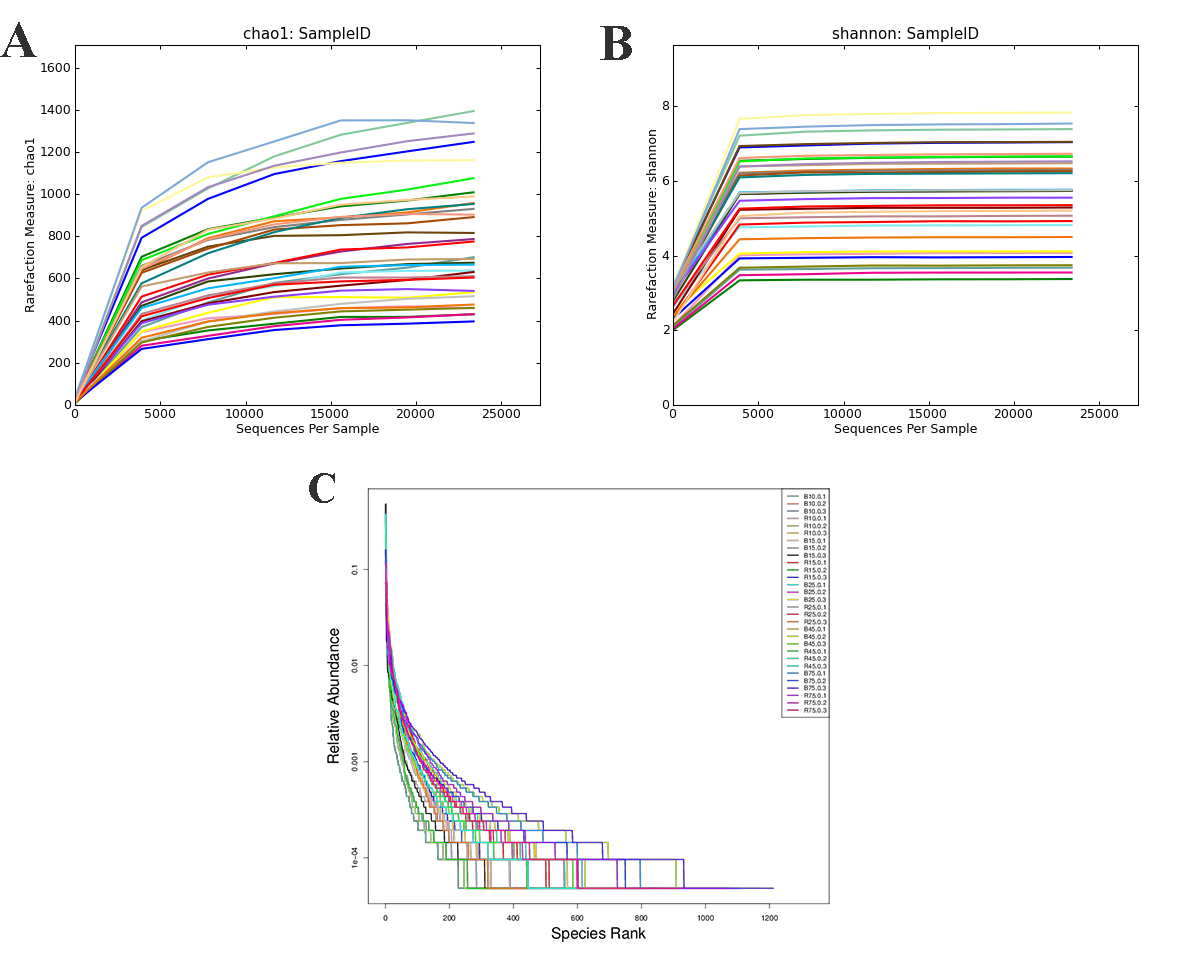

Supplement: Supplementary Figure 1 — Feasibility analysis of different samples. Each curve represents a sample. The rarefaction curves (A,B) were used to evaluate the adequacy of sequencing for each sample and the Rank abundance curve (C) was used to assess the abundance and evenness of samples. B represents the YBGs in the control group, while R indicates the YBGs in the milk replacer supplementation group. [file Image_1.tif]
